# Does perceived caregiver HIV stigma and depression increase adolescent neuro-behavioral difficulties? A mediation analysis in the Asenze Cohort

**DOI:** 10.21203/rs.3.rs-4543382/v1

**Published:** 2024-07-15

**Authors:** Amaleah F. Mirti, Jeremy C. Kane, Kathryn G. Watt, Chris Desmond, Rachel S. Gruver, Adele Munsami, Nonhlanhla P. Myeza, Gabriela A. Norwitz, Leslie L. Davidson

**Affiliations:** 1 Department of Sociomedical Sciences, Columbia University, New York City, USA; 2 Department of Epidemiology, Columbia University, New York City, USA; 3 Centre for Rural Health, School of Nursing & Public Health, University of KwaZulu-Natal, Durban, South Africa; 4 School of Economics and Finance, University of the Witwatersrand, Johannesburg, South Africa

**Keywords:** HIV stigma, caregiver, adolescent neuro-behavioral difficulties, cohort study, LMIC, mediation

## Abstract

People living with HIV (PLWH) often experience HIV related stigma that is, in turn, associated with several negative health outcomes including depression, harmful drinking, and intimate partner violence. Despite knowledge of these proximal impacts of HIV stigma on PLWH, less is known about the impact that Caregivers living with HIV’s perception of stigma has on the health and behavior of adolescents in their care.

Utilizing data from adolescents and their primary caregivers from the population-based Asenze cohort study in KwaZulu-Natal, South Africa, we conducted a path analysis to determine if caregiver depression [operationalized as mental health functioning] is a mediator of the hypothesized association between caregiver HIV stigma and adolescent neurodevelopmental behavior including internalizing and externalizing behaviors.

Results suggest good model fit and a statistically significant relationship between caregiver HIV stigma and caregiver mental health functioning. However, neither the direct nor indirect (including potential mediator caregiver mental health functioning) effect of HIV stigma on adolescent behavioral difficulties was statistically significant.

This paper builds on previous research demonstrating the relationship between HIV stigma and depression, highlighting the need for continued study of underlying mechanisms that impact the stigma and health of PLWH and others important to them such as their children.

## Introduction

People living with HIV (PLWH) often experience stigma that contributes to many negative health and social outcomes, such as depression, harmful drinking, and intimate partner violence (Fiorenino et al., 2019; Fisher et al., 2007; Jones et al., 2020). In some regions, the issue of HIV stigma is especially critical: South Africa has the largest number of PLWH globally (7.5 million) and in the province of KwaZulu-Natal (KZN), 21% of the population are currently living with HIV (Kim et al., 2021; Pettifor et al., 2003). It is important to understand both the ongoing presence of stigma and the mechanisms by which HIV stigma impacts PLWH and their families.

Goffman defines stigma as an attribute that is considered undesirable by elements of society (Goffman, 1986). According to Link and Phelan, stigma occurs when labeling, stereotyping, discrimination, loss of status, and separation interact in the context of unequal power dynamics between those stigmatized and society (Link & Phelan, 2001).

The detrimental effects of HIV stigma on the health of PLWH are well studied in both adults and children globally (Jones et al., 2020; Mojola et al., 2022). A meta-analysis of 24 studies located primarily in the United States but with representation from Sub-Saharan Africa (SSA), Asia, and Europe found a moderate correlation between HIV stigma and depression symptoms (Rueda et al., 2016). Several studies in SSA have established an association between HIV stigma and depression with increasing evidence for internalized stigma leading to increased depression severity in both adults and adolescents living with HIV in South Africa (Babor et al., 2001; Bebell et al., 2021; Breet et al., 2014; Kalichman et al., 2009; MacLean & Wetherall, 2021; Williams et al., 2019; Wouters et al., 2016).

HIV stigma has also been found to contribute to alcohol misuse. A longitudinal study in Ontario, Canada found that experiencing HIV stigma contributes to reliance on maladaptive coping strategies such as alcohol consumption, and noted that the relationship between HIV stigma, coping, and alcohol use may be bidirectional (Wardell et al., 2018). [17] A systematic review of alcohol use in LMICs posits that household alcohol use is associated with negative adolescent behavioral outcomes (Jokinen et al., 2021).

Experiencing HIV stigma is also associated with intimate partner violence (IPV). A study in Cameroon found that women living with HIV reporting high HIV-related stigma had 2.53 times the odds of experiencing frequent physical IPV, compared to women that did not report HIV-related stigma (Fiorenino et al., 2019). A mixed-methods study in the United States found that HIV stigma was the most reported reason that PLWH stayed in an abusive relationship (Davis et al., 2021). Reported reasons for remaining in the abusive relationship included their partner’s willingness to remain with them despite their HIV status, fear of their status being disclosed should they leave the relationship, and fear of rejection in another relationship (Davis et al., 2021). Additionally, in the United States, men and women with past experiences of IPV report riskier sexual behaviors that increased their risk for contracting HIV (Breiding et al., 2008). Given the complexity and bidirectional relationship between HIV stigma and IPV, it is crucial to understand HIV stigma in the context of IPV in SSA (Yonga et al., 2022).

In addition to the association in adults between HIV stigma and depression, alcohol use, and IPV, these factors are also known to contribute to neuro-behavioral difficulties in the children and adolescents they care for. The association between caregiver depression and child neuro-behavioral outcomes has been studied in several contexts (Meyer et al., 2017; Netsi et al., 2018; Weissman et al., 1997). In a U.S.-based study caregiver depression scores were positively associated with an increase in child behavioral problems, both internalizing and externalizing (Levin et al., 2022). Children living in environments with exposure to adverse childhood experiences (ACEs) such as caregiver mental illness or experience of IPV have worsened mental and behavioral outcomes as adolescents (Garrido et al., 2018; Jokinen et al., 2021). In the Asenze cohort study, which supplied data for this paper’s analysis, a positive relationship was found between the number of ACEs measured at age five and children’s neurobehavioral outcomes at average age seven (Nazareth et al., 2022).

Caregiver IPV and alcohol misuse have also been linked with child behavior difficulties in our study’s cohort. In the first wave of the Asenze study, caregivers’ experience with IPV was associated with child behavior problems even after adjusting for caregiver binge drinking, caregiver depression, and child HIV status (Chander et al., 2017). In the second wave, caregiver hazardous drinking was positively associated with child problem behavior at average age seven, even after adjusting for child age, gender, and HIV status as well as caregiver type, HIV status, PTSD and household asset index (Azevedo Da Silva et al., 2022).

Some evidence suggests that HIV stigma may contribute to mental health difficulties among children whose caregivers are living with HIV, regardless of the child’s own HIV status (Betancourt et al., 2014). A study in Rwanda looked at the relationship between HIV-affected children (defined as children whose caregivers lived with HIV) and child mental health (Betancourt et al., 2014). This study found that rates of mental health problems among HIV-affected children were similar to those who were living with HIV themselves (Betancourt et al., 2014). They also found similar levels of stigma among children living with HIV and children affected by caregiver HIV and proposed that HIV stigma experienced by both groups of children could be an underlying mechanism contributing to their mental health problems (Betancourt et al., 2014).

**Figure 1:**
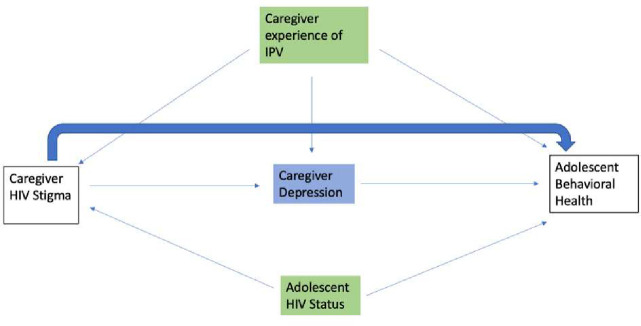
Conceptual Diagram.

This paper will expand upon the findings of the Rwandan study and examine the effect of stigma experienced by a caregiver living with HIV on the neuro-behavioral difficulties of an adolescent living in their same household. Due to the multifaceted proximal outcomes resulting from HIV stigma, we examine both caregiver depression and alcohol abuse as potential mediators of the relationship between caregiver HIV stigma and adolescent neuro-behavioral difficulties (Figure 1).

Using data from caregivers living with HIV and the adolescents they look after in the Asenze cohort, this paper aims to:

1. Examine the impact of HIV stigma experienced by primary caregivers on adolescent behavioral difficulties
2. Explore the extent to which any relationship between caregiver-experienced HIV stigma and adolescent behavior is mediated by caregiver depression and binge/risky drinking.

## Methods

### I. Study Procedure and Characteristics of Participants

The Asenze Cohort in KZN, South Africa is comprised of caregivers and young people living in a peri-rural area. In the first wave, children aged 4–6 years old were identified as part of a population-based door-to-door survey between 2008 and 2010 (Chhagan et al., 2014; Desmond et al., 2022). Wave 3 assessments were completed between 2019 and 2021. Adolescent questionnaires were self-administered on tablets in either English or Zulu and caregiver questionnaires were administered verbally in Zulu by trained mid-level assessors who recorded responses.

**Figure 2:**
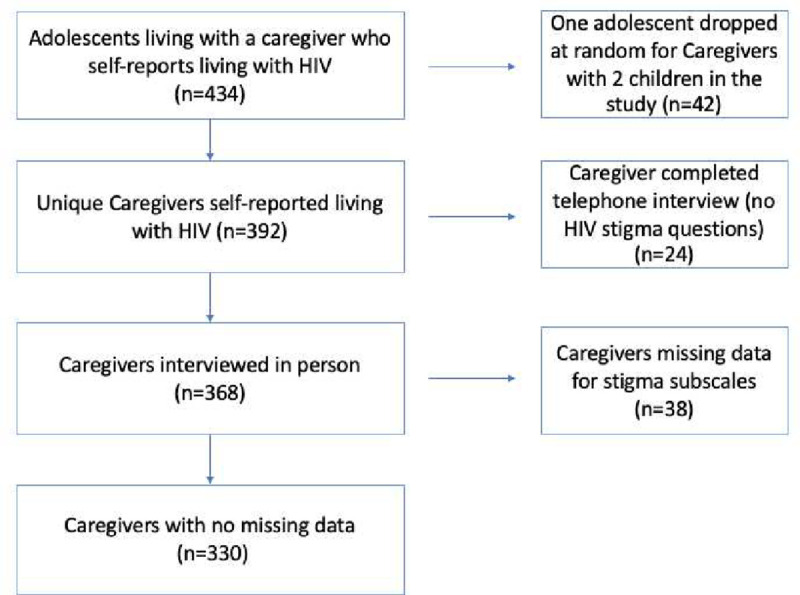
Sample Exclusion.

Wave 3 data collected when the study children (n=1,201) were adolescents (age range: 13–19 years, average: 15.9) was used for this analysis. In this cohort, 392 of the caregivers acknowledged living with HIV, and therefore completed an HIV stigma questionnaire. These 392 caregivers and the 434 adolescents under their care were eligible for this analysis. In the sample, 42 caregivers each looked after two participating children. To address potential family-level clustering in the model, one of the two children under the same caregiver was randomly dropped from the sample making a total of 392 caregiver-adolescent dyads in this analysis (Figure 2).

Informed written consent was obtained from each research participant, their adult caregiver participant, or a legally authorized representative. The caregiver was asked to consent for their own participation, as well as their adolescent child’s. The adolescent was invited to assent. Additional consent for HIV testing was requested, but was not required for participation in the study. The consent forms were read aloud and a copy was given to the participants. Due to the pandemic some participants were assessed telephonically; therefore, informed consent was obtained with an approved waiver of written documentation. Both the Columbia University Institutional Review Board and the University of KwaZulu-Natal Biomedical Research Ethics Committee approved Wave 3 of the Asenze study.

### II. Measures Used

All study tools were translated from English into Zulu and back-translated using a modified committee approach. All committee members were experienced bilingual assessors in English and Zulu (Desmond et al., 2022).

#### Caregiver HIV stigma:

Berger et al.’s 40-item HIV stigma Scale is a widely used measure globally and includes subscales of: personalized stigma (enacted stigma), disclosure concerns (anticipated stigma), negative self-image (internalized stigma), and concern with public attitudes about people with HIV (anticipated stigma) (Berger et al., 2001; Earnshaw & Chaudoir, 2009). A short 12-item version developed by Reinus et al. of Berger’s scale was used in the Asenze study as it has shown comparable psychometric properties (Reinius et al., 2017). The 12-item scale was comprised of the same four subscales: personalized stigma, disclosure concerns, concerns about public attitudes, and negative self-image (Reinius et al., 2017). (Saunders)A total HIV stigma score was calculated by summing the responses to each of the questions; a higher number corresponding to higher levels of stigma. This total score encompasses all the subscales and hypothesized mechanisms. Caregivers that had missing data for any of the questions or subscales were dropped from the analysis (Figure 2). The sub-scale on personalized stigma was not administered to those who had not disclosed their status to others (n=38), without a complete measure of HIV stigma they were excluded from the analysis.

#### Adolescent Behavioral Difficulties:

The Strengths and Difficulties Questionnaire (SDQ) consists of five subscales: Emotional Symptoms, Conduct Problems, Hyperactivity/Inattention, Relationship Problems, and Prosocial Behavior (Goodman, 1997; Mind, 2016). The SDQ self-report, for children and adolescents 11 years and older, was used in Asenze wave 3 and has been used in the African continent with adequate internal consistency (Goodman, 1997; Hoosen et al., 2018) The SDQ parent-report version was used in the first two waves of the Asenze cohort when the children were on average 5 and 7 years old. An assessment of its psychometric properties in this population found that the Total Difficulties score had a satisfactory internal consistency of 0.74 (Mellins et al., 2018). The Total Difficulties score was used as the measure of adolescent behavioral difficulties for this analysis. Though the SDQ questionnaire was administered to both the adolescent and reported by the caregiver, only the adolescent’s self-report was used for the analysis, as the caregiver’s HIV status, level of stigma and depression could affect caregiver report about the adolescent’s behavior.

#### Caregiver Unhealthy Drinking:

Unhealthy drinking was measured using the WHO Alcohol Use Disorders Identification Test (AUDIT) which has been used in all three waves of Asenze as well as in other studies conducted in South Africa (Azevedo Da Silva et al., 2022; Taylor et al., 2016). This paper uses the World Health Organization cutoffs of the AUDIT (Babor et al., 2001). A score of 0 indicates an individual who abstains from drinking alcohol (Saunders). Upon review of the data, we found that 79% of caregivers in the eligible sample reported abstaining from any drinking, while only 5% had an AUDIT score in a high-risk category (≥8). As there were too few caregivers that met the criteria for “any drinking”, this variable was dropped from the path model as a potential mediator.

#### Caregiver Mental Health:

The RAND 36-Item Health Survey (Medical Outcomes Study 36-item Short Form Health Survey) Mental Health component score is comprised of 4 domains: role limitations due to personal or emotional problems, emotional well-being, social functioning, and energy/fatigue (Ware & Sherbourne, 1992). The RAND-36 scoring method was used to calculate the mental component score measuring depression in the caregiver, with higher scores indicating better quality of mental health (Anagnostopoulos et al., 2009; Hays et al., 1995).

#### Caregiver Intimate Partner Violence:

Each caregiver was asked if they had ever had a partner, and subsequently if they currently had a partner. The caregiver was asked about their experience of physical IPV (“has your current partner/any previous partner ever pushed or shoved you…ever slapped you or threw something at you?”), sexual IPV (“has your current partner/any previous partner every physically forced you to have sex?”), and threatening IPV (“I am afraid of my current partner” or “have any of your previous partners ever threatened to hurt you?”). Their responses to these questions were coded as a binary variable where 1= participants experienced any form of IPV from a current/previous partner and/or feel threatened by a current/previous partner, and 0 = none of the above. From these multiple binary variables, a composite IPV variable was constructed where 1=ever experienced any IPV, and 0 = no IPV.

IPV was included in the model due to its high prevalence within the caregiver population in the Asenze cohort. In addition, an earlier study on wave 1 of the Asenze cohort found that caregiver IPV experience was associated with child behavioral difficulties (Chander et al., 2017). Therefore, IPV was considered a confounder (Figure 1) as opposed to an effect modifier due to this prior association as well as additional literature establishing evidence of a relationship between IPV and HIV stigma.

#### Adolescent HIV Status:

All adolescents in the sample for this paper had a caregiver living with HIV. However, some adolescents themselves were living with HIV, predominantly perinatally acquired. Adolescent HIV status was determined by self-report and corroborated with testing data where available. Adolescent HIV status was considered a confounder in the path model because of established associations with potential upstream predictors of poorer adolescent behavior. Adolescents from the entire Asenze cohort living with perinatal HIV had increased prevalence of stunted growth, hearing impairment, food insecurity, and cognitive and language delay (Gruver et al., 2020; Knox et al., 2018). In other contexts, caregivers of children living with HIV report concern for their child’s status becoming known to others and their child facing subsequent discrimination (McHenry et al., 2017; Sarkar et al., 2018).

### III. Analysis

First, the distributions of caregiver HIV stigma, total adolescent behavior difficulties, and caregiver depression, IPV, and unhealthy drinking were examined. Then, based on the conceptual model (Figure 1) a path model was estimated to test the exploratory mediation hypothesis using M*plus* (Muthén & Muthén, 1998). Caregiver HIV stigma was the exposure, and adolescent behavioral difficulty was the primary outcome of interest. Caregiver mental health and unhealthy drinking were the hypothesized mediators; caregiver experience of IPV, and adolescent HIV status were controlled for as potential confounders. As caregiver gender only confounded the pathway between HIV stigma and the mediator (caregiver depression), it was not included in the path model. Analysis was conducted using Stata, version 15 and M*plus*, version 8 (Muthén & Muthén, 1998; StataCorp, 2017).

## Results

Of the 1,044 caregivers in wave 3 of the Asenze study, 1,008 said that they had been tested for HIV and 392 (36%) self-reported living with HIV. Of these 392 caregivers, 94% (n=369) identified as female. Once caregivers who did not have complete data on the HIV stigma variable were excluded, the total number of caregivers was 330 (96% female, n=315). The mean age of caregivers in the analytic sample was 35.6 years old, with the entire sample ranging from 18 to 71 years old. In the analytic sample, 71% (n=233) of the caregivers were the adolescent’s biological parent, and 28% were relatives such as an aunt or grandparent. Sixty-eight percent (n=225) of caregivers in the sample reported currently having a partner, and 59% (n=196) had a partner(s) in the past. Overall, 52% (n=172) caregivers had experienced some form of IPV at some point with a past or current partner (Table 1).

Of the 330 adolescents included in the analysis 52% (n=173) were female, and two adolescents had no gender recorded. The average age of the adolescents was 15.9 years. In the analytic sample of 330 adolescents, 18 (just over 5%) reported their HIV status as positive. Only 12% (n=124) caregivers in the entire cohort accepted HIV testing at wave 3, of those 124, only one individual tested positive but self-reported as HIV negative.

The fit statistics provided in Table 2 suggest that the model is a good fit for the data.

In this analysis Caregiver HIV Stigma significantly predicted caregiver mental health (*β* =−0.42, p-value = 0.007). Additionally, adolescent HIV status significantly predicted adolescent behavioral difficulties (*β* =3.95, p-value= 0.008). There was a marginally statistically significant finding of caregiver mental health predicting adolescent behavioral difficulties (*β* =−0.04, p-value = 0.09). Neither the direct nor indirect effect of HIV stigma on adolescent behavioral difficulties as mediated through caregiver mental health was statistically significant.

## Discussion

The Asenze study, using a socio-ecological model over the lifecourse, aims to understand how experiences in childhood and adolescence can shape behavior, development, and health in a context of high HIV prevalence. This paper explored one potential mechanistic pathway between caregiver HIV stigma and adolescent behavior, investigating whether caregiver mental health mediated this relationship. The results did not suggest a significant relationship between caregiver HIV stigma and adolescent neuro-behavioral difficulties, thus caregiver mental health as measured does not appear to mediate this relationship in this sample of the Asenze study population. However, we did find a significant association between caregiver HIV stigma and caregiver mental health. This finding aligns with prior research in South Africa establishing a relationship between HIV stigma and caregiver depression (Bebell et al., 2021; Breet et al., 2014; Simbayi et al., 2007; Wouters et al., 2016). While not statistically significant, there was a trend between poor caregiver mental health and adolescent behavior difficulties: as caregiver mental health composite score decreased, the total difficulties score on the adolescent SDQ increased. The relationship between caregiver mental health and adolescent behavioral difficulties is well documented in the literature, but not in populations where the HIV burden of caregivers is high (Laurenzi et al., 2021; Panter-Brick et al., 2014; Simbayi et al., 2007). This difference, along with our sample size may explain why a significant relationship was not observed.

Limitations of the paper: First, because the analytic sample within the Asenze cohort was restricted to PLWH, the sample size was limited thus reducing statistical power which may have contributed to null findings. Another important limitation is that we cannot clearly establish temporality between the onset of caregiver experience of HIV stigma, their mental health, and the measure of the adolescent’s behavioral difficulties. Available longitudinal data provide some insights into temporality; of all caregivers assessed at Wave 3, 166 were living with HIV at wave 1, and 225 were living with HIV at wave 2. Therefore, over half of the initial sample of 392 caregivers have been living with HIV since 2012. This establishes temporality between the proposed exposure and mediator, for more than half of the sample.

The SF-36 asked about experiences within the past four weeks, which roughly establishes temporality for the remainder of the sample. However, establishing temporality between exposure/mediator and adolescent neuro-behavioral difficulties is not possible, and made more complicated by the fact we do not know how long the adolescent has been living with the caregiver (for example: in the event of death or other circumstances, caregivers, unlike the adolescents, could change between study waves). Thus, we are potentially equating adolescents who have been with a caregiver for their entire life, versus those that have had their caregivers change. This could obscure associations between both caregiver depression and caregiver HIV stigma and adolescent behavioral difficulties. Additionally, given that we are looking at an adolescent-caregiver relationship compared to a young child-caregiver relationship the impact of caregiver depression and/or HIV stigma on adolescent behavioral difficulties may be less apparent, potentially explaining the null findings.

Aside from issues with temporality there is also potential selection bias. Only those caregivers that self-reported living with HIV were administered the HIV stigma questionnaire. Caregivers with high levels of perceived stigma may have been unwilling to disclose their HIV status to the study, and subsequently missing from our sample. Additionally, the individuals with missing values on the subscale for disclosure concerns excluded from the analysis had, on average, statistically significant (p <0.001) higher scores on the other stigma subscales than those individuals who had not marked “Not Applicable”. This indicates that these caregivers with missing HIV stigma data are somewhat different than those without the missing data, and thus our analytic sample likely has lower HIV stigma scores than the population of all Asenze caregivers living with HIV. It is however unlikely that these exclusions would have changed the direction of the relationships but they may have affected the power statistically.

This paper’s strengths include the use of measures validated within this population and the unique setting of KZN, a province with one of the highest HIV infection rates globally. The SDQ has been validated in this population’s children at a prior wave, lending strength to the inferences drawn (Mellins et al., 2018). Examining the impact of caregiver HIV stigma within a population-based cohort study allows one to understand the burden of HIV to the collective society as opposed to a clinical sample; the Asenze cohort study enrolled a high proportion of the eligible children and their caregivers living in the study area. As experiences of stigma are largely dependent on social context, this study elucidates the experience of HIV stigma among caregivers in an area of high HIV prevalence.

## Conclusion

This study establishes, within the context of the population-based Asenze cohort, a clear association between caregivers’ experience of HIV stigma and their mental health functioning. This finding bolsters findings from other studies in KZN, as well as South Africa at large, on the relationship between HIV stigma and mental health for PLWH. Moreover, it highlights the importance of understanding HIV from a psychosocial perspective and reaffirms the need for mental health resources in populations with large numbers of people living with HIV. It also underscores the need to develop interventions that reduce stigma and that target and disrupt the mechanisms between HIV stigma impact on mental health. While this analysis did not find a significant association between caregiver perception of HIV stigma and the neuro-behavioral difficulties reported by adolescents under their care, this relationship could still exist undetected by this analysis due to insufficient power. More research should be done to understand how the transition to adolescence and early adulthood may mask the importance of caregiver mental health on neurobehavioral difficulties. To further understand the relationship between the impact of HIV infection and mental health problems in caregivers on adolescent health outcomes, it is important to not only assess HIV status in studies, but also stigma.

## Figures and Tables

**Figure 3:**
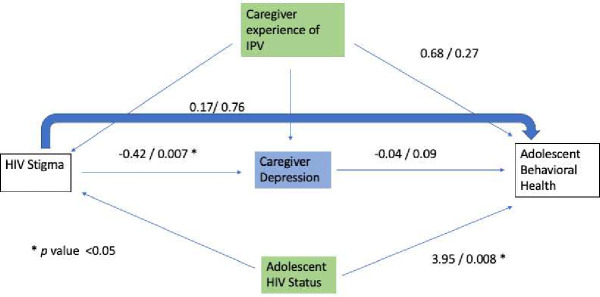
Path model with estimated coefficients.

**Table 1:** Analytic Study Sample Participant Characteristics

Characteristic	N = 330
**Adolescent Gender** ^1^	
Female	173(52.4%)
Male	155(47.0%)
Not Reported	2(0.6%)
**Caregiver Gender** ^1^	
Female	315(95.5%)
Male	15(4.5%)
**Adolescent Age** ^2^	15.89 (0.9)
**Caregiver Age** ^2, 6^	35.65 (9.5)
**Total HIV Stigma Score** ^2, 3^	25 (5.8)
**SDQ total Adolescent Difficulty Score (continuous)** ^2, 4^	13.8 (6.1)
**Ever experienced IPV** ^1^	172 (52.1%)
**Mental Composite Score SF-36** ^2, 5^	79 (16.5)
**Adolescents Living with HIV** ^1^	18(5.5%)

1 n(%)

2 Mean (SD)

3 Score range (12–48)

4 SDQ score range (0–40)

5 SF36 Range (0–100)

6 n=71 had age missing

**Table 2:** **Path Model fit indices** (N=330)

Index	Model Value	Reference threshold suggesting good fit
*χ* ^2^	2.89 (*p*=0.24)	*P* > 0.05
**CFI**	0.94	> 0.90
**RMSEA**	0.037	<0.08
**SRMR**	0.021	<0.08

The path coefficients are presented in both Table 3 and in Figure 3.

**Table 3:** Estimated path coefficients

Path	Estimate (unstandardized)	Standard Error	p	Estimate (standardized)
**Direct Effects**				
**HIV stigma ➔ adolescent neuro-behavioral difficulties**	0.02	0.06	0.76	0.003
**HIV stigma ➔ caregiver mental health**	−0.42	0.16	0.007	−0.03
**caregiver mental health ➔ adolescent neuro-behavioral difficulties**	−0.04	0.02	0.09	−0.09
**IPV ➔ adolescent neuro-behavioral difficulties**	0.77	0.69	0.27	0.12
**Adolescent HIV Status ➔ adolescent neuro-behavioral difficulties**	3.95	1.48	0.008	0.64
**Indirect Effects**				
**HIV stigma ➔ caregiver mental health ➔ adolescent neuro-behavioral difficulties**	0.02	0.01	0.16	0.02

## Data Availability

The data that support the findings of this study are available on request and with ethics approval from the corresponding author Dr. Kane, and MPIs Dr. Davidson and Dr. Desmond.
